# ProCARs: Progressive Reconstruction of Ancestral Gene Orders

**DOI:** 10.1186/1471-2164-16-S5-S6

**Published:** 2015-05-26

**Authors:** Amandine Perrin, Jean-Stéphane Varré, Samuel Blanquart, Aïda Ouangraoua

**Affiliations:** 1 Inria Lille Nord-Europe, LIFL UMR CNRS 8022, Université Lille 1, Lille, France; 2 Département d'informatique, Université de Sherbrooke, Sherbrooke, Canada

**Keywords:** Ancestral gene orders reconstruction, Small phylogeny problem, Boreoeutherian ancestor

## Abstract

**Background:**

In the context of ancestral gene order reconstruction from extant genomes, there exist two main computational approaches: rearrangement-based, and homology-based methods. The rearrangement-based methods consist in minimizing a total rearrangement distance on the branches of a species tree. The homology-based methods consist in the detection of a set of potential ancestral contiguity features, followed by the assembling of these features into Contiguous Ancestral Regions (CARs).

**Results:**

In this paper, we present a new homology-based method that uses a progressive approach for both the detection and the assembling of ancestral contiguity features into CARs. The method is based on detecting a set of potential ancestral adjacencies iteratively using the current set of CARs at each step, and constructing CARs progressively using a 2-phase assembling method.

**Conclusion:**

We show the usefulness of the method through a reconstruction of the boreoeutherian ancestral gene order, and a comparison with three other homology-based methods: AnGeS, InferCARs and GapAdj. The program, written in Python, and the dataset used in this paper are available at http://bioinfo.lifl.fr/procars/.

## Background

The small phylogeny problem consists in reconstructing the ancestral gene orders at the internal nodes of a species tree, given the gene orders of the extant genomes at the leaves of the tree. There exist two main computational approaches for the reconstruction of ancestral gene orders from extant gene orders: rearrangement-based methods, and homology-based methods.

The rearrangement-based methods require a rearrangement model, and consist in finding a rearrangement scenario that minimizes the total rearrangement distance on the branches of the species tree [1-3]. The homology-based methods consist in finding the ancestral gene orders associated with the internal nodes of the species tree, such that the total amount of homoplasy phenomenon observed in the species tree is minimized [4-9]. Homoplasy is a phenomenon by which two genomes in different lineages acquire independently a same feature that is not shared and derived from a common ancestor. For the inference of the ancestral gene order at a tagged internal node, the homology-based methods are usually composed of two steps. The first step consists in detecting a set of potential ancestral contiguity features, by comparison of pairs of extant genomes whose path goes through the ancestor in the species tree. The second step is an assembling phase that requires to compute an accurate conservation score for each potential ancestral feature, based on the species tree. Using these scores, some heuristic algorithms are then used to resolve the conflicts between the ancestral features in order to assemble them into Contiguous Ancestral Regions (CARs). A *CAR *of an ancestral genome is an ordered sequence of oriented blocks (genes, or synteny blocks) that potentially appear consecutively in this ancestral genome.

In the absence of tangible evolution model, the homology-based methods have the convenience to reconstruct CARs that contain only reliable features inferred from a conservation signal observed in the extant genomes. However, the ancestral genomes reconstructed using homology-based methods are often not completely assembled, as some rearrangement or content-modifying events might have caused the loss of some ancestral contiguity features in the extant genomes. Thus, the homology-based methods proposed in the literature usually enlarge the condition of contiguity in order to detect more potential ancestral contiguity features, -adjacencies between two blocks [5,6,9], maximum common intervals of blocks [4,10,7], gapped adjacencies [11]. Hence, these different types of contiguity features can be classified according to the tightness of their definition of contiguity. The homology-based methods should then account for this classification when assembling different types of contiguity features. This approach was used in [11] where a method, GapAdj, was presented for iteratively detecting gapped adjacencies. GapAdj uses a progressively relaxed definition of contiguity allowing an increasing number of gaps between ancestral contiguous synteny blocks in extant genomes, and iteratively assembling these gapped adjacencies using a heuristic Traveling Salesman algorithm (TSP). The TSP is applied on a graph whose vertices are synteny blocks, and edges are potential ancestral adjacencies between these blocks.

Here, we follow the same idea, and we present an homology-based method that is based on *iteratively *detecting and assembling ancestral adjacencies, while allowing some micro-rearrangements of synteny blocks at the extremities of the progressively assembled CARs. The method starts with a set of non-duplicated blocks as the initial set of CARs, and detects iteratively the potential ancestral adjacencies between extremities of CARs, while building up the CARs *progressively *by adding, at each step, new non-conflicting adjacencies that induce the less homoplasy phenomenon. The species tree is used, in some additional internal steps, to compute a score for the remaining conflicting adjacencies, and to detect other reliable adjacencies, in order to reach completely assembled ancestral genomes. The first originality of the method comes from the usage of the progressively assembled CARs for the detection of ancestral contiguity features allowing micro-rearrangements. The second originality comes from the assembling method at each iterative step that consists in adding the contiguity features gradually giving priority to the features that minimize the homoplasy phenomenon, rather than relying on a heuristic algorithm for discarding false-positive features. We discuss the usefulness of the method through a comparison with three other homology-based methods (AnGeS [12], InferCARs [5] and GapAdj [11]) on the same real dataset of amniote genomes for the reconstruction of the boreoeutherian genome.

## Preliminaries: genomes, species tree, conserved adjacencies

For the reconstruction of ancestral genomes from extant ones, genomes are represented by identifying homologous conserved segments along their DNA sequences, called *synteny blocks*. These blocks can be relatively small, or very large fragments of chromosomes. The order and orientation of the blocks, and their distribution on chromosomes may vary in different genomes. A *signed block *is a block preceded by a sign + or − representing its orientation. By convention, a signed block +*a *is simply written *a*. Here we assume that all *genomes *contain the same set of non-duplicated blocks and consist of several circular or linear chromosomes composed of signed blocks.

**Figure 1:**
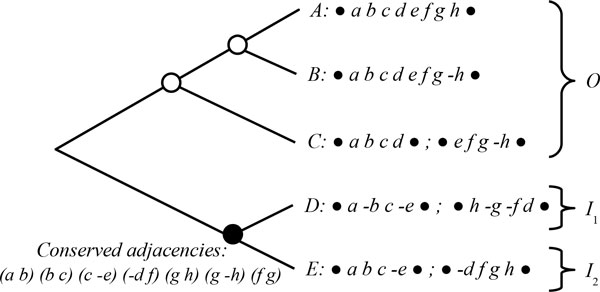
**Example of a species tree**. A species tree on five genomes *A*, *B*, *C*, *D*, and *E*. The black-colored ancestral node defines two ingroup sets each composed of a single genome, *I*_1 _= {*D*} and *I*_2 _= {*E*}, and an outgroup set *O *= {*A, B, C*}. The conserved adjacencies at the ancestral black-colored node are given.

For example, consider the five genomes represented at the leaves of the tree in Figure 1. The bullets at the extremities of the chromosomes represent the telomeres of linear chromosomes. Genomes *A *and *B *consist of one linear chromosome each, and genomes *C*, *D*, and *E *consist of two linear chromosomes each.

A *Contiguous Ancestral Region (CAR) *is defined as a potential chromosome of an ancestral genome.

A *segment *in a genome is an ordered set of signed blocks that appear consecutively in the genome. The *length *of a segment is the number of blocks composing this segment. In the above example, {*b c d e*} is a segment of length 4 in the genome *A*.

Two segments of two different genomes are called *syntenic segments *if they contain the same set of blocks. For example, the segments {*h −g − f d*} of genome *D *and {*−d f g h*} of genome *E *are syntenic.

An *adjacency *in a genome is an ordered pair of two consecutive signed blocks. For example, in the above genomes, (*a b*) is an adjacency of genomes *A*, *B*, *C*, and *E*, and (*a −b*) is an adjacency of genome *B*. Since a whole chromosome or a segment can always be flipped, we have (*x y*) = (*−y −x*). For example, (*g −h*) = (*h −g*) is an adjacency shared by genomes *B*, *C*, and *D*.

A *species tree *on a set of *k *genomes is a rooted tree with *k *leaves, where each genome is associated with a single leaf of the tree, and the internal nodes of the tree represent ancestral genomes. For example, Figure 1 shows a species tree on genomes *A*, *B*, *C*, *D*, and *E*.

Here, for the reconstruction of ancestral gene orders, we consider an ancestral node of the species tree that has exactly two children resulting from a speciation (black-colored node in Figure 1). The *species partition defined by an ancestral node *is the partition of the extant species into three sets: two ingroup sets *I*_1 _and *I*_2 _corresponding to the two lineages descending from the ancestor, and one outgroup set *O *containing all other extant genomes.

A *conserved adjacency *at an ancestral node of the species tree is an adjacency shared by at least two genomes belonging to at least two different sets of the species partition defined by the ancestral node. Such two genomes are linked by a path that goes through the ancestral node. For example, in Figure 1, (*a b*) is a conserved adjacency of the black-colored ancestral node because it is shared by genomes *C *and *E *whose path goes through the ancestor. The adjacency (*c −e*) is also a conserved adjacency of this ancestor because of its presence in genomes *D *and *E*.

A conserved adjacency at an ancestral node is considered as a potential adjacency of this ancestor. Homology-based methods for the reconstruction of ancestral gene orders usually consist in, first, detecting all the conserved adjacencies at the ancestral node, and next, assembling these conserved adjacencies into CARs. The difficulty in this assembling phase comes from the conflicts that may exist between some conserved adjacencies. Two adjacencies are called *conflicting adjacencies *when they involve a same block extremity, and thus they cannot be both present in the same ancestral genome. For example, in Figure 1, the conserved adjacencies (*g h*) and (*g −h*) of the black-colored node are conflicting as they both involve the right extremity of block *g*. Two adjacencies that are not conflicting are called *compatible*. A set of adjacencies is said *non-conflicting (NC) *if all pairs of adjacencies in the set are compatible.

Here, we distinguish two types of conserved adjacency regarding their presence or absence in the three sets of species defined by the considered ancestral node: the two ingroup sets *I*_1 _and *I*_2_, and the outgroup set *O*. A *fully-conserved adjacency *is a conserved adjacency that is present in at least one genome of each of the three sets of species. A *partly-conserved adjacency *is any other conserved adjacency. For example, in Figure 1, (*f g*) is a fully-conserved adjacency of the black-colored ancestral node, while all other conserved adjacencies are partly-conserved adjacencies.

The *homoplasy cost *of an adjacency at a given ancestral node *A *counts the number of branches linked to this ancestor on which the adjacency would have been gained (right before the ancestor) or lost (after the ancestor) if it was present in the ancestor. It is defined as follows: it is either 0 if the adjacency is fully-conserved at *A*, or 1 if it is partly-conserved at *A*, or 2 if it is present in only one of the sets *I*_1_, *I*_2 _and *O*, or 3 if it is present in none of these sets. Note that if an adjacency has an homoplasy cost of 2 or 3 at the ancestral node *A*, then the adjacency is not conserved at this node. For example, in Figure 1, the adjacency (*f g*) has a cost 0, the adjacency (*a b*) a cost 1, the adjacency (*a −b*) a cost 2, while the adjacency (*a c*) has a cost 3.

## Method

The homology-based problem considered in this paper for the reconstruction of ancestral gene orders can be stated as follows:

*Problem*. Given a species tree on a set of extant genomes, each composed of the same set of blocks, and given an ancestral node in this species tree, find a set of CARs at the ancestral node, with a maximum number of adjacencies, that minimizes the total homoplasy cost.

Compared to other homology-based methods for the reconstruction of ancestral gene orders, the progressive method presented in the following consists in adding adjacencies progressively, as opposed to discarding false adjacencies in a single assembling step.

A global description of the progressive method steps is presented in the following, and the refined descriptions are presented next.

### Inputs and start

The input of the method is a phylogeny with a tagged ancestral node whose block order is to be reconstructed, and a set of *n *orthologous blocks that are used to describe the block orders of the genomes at the leaves of the tree. The initialization of the method consists in starting with an initial set of *n *CARs, each composed of a single block.

### Overall idea

**Figure 2:**
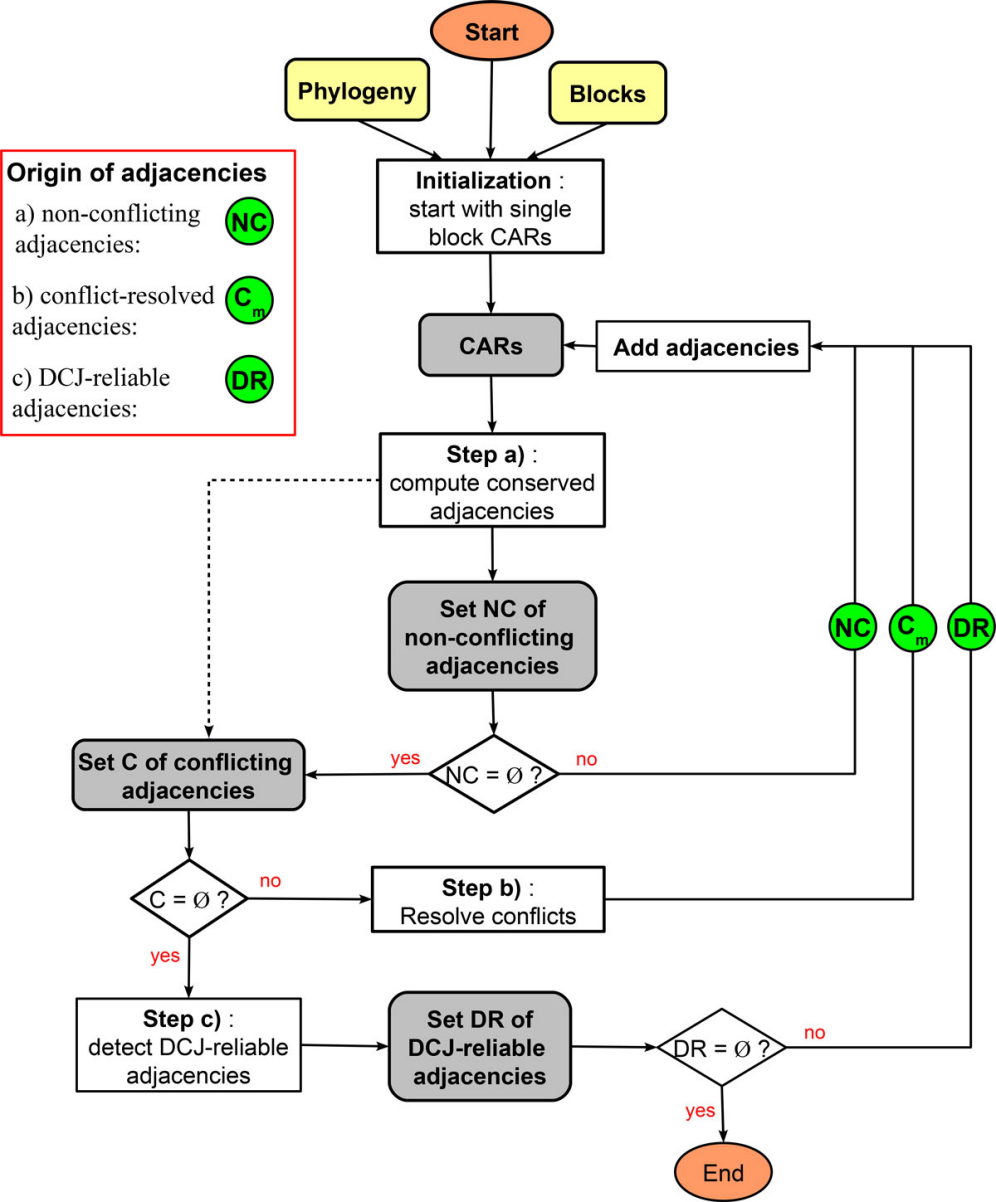
**Diagram of the method steps**. Overall description of the ProCARs method steps

The core of the method relies on iteratively computing new block adjacencies in order to concatenate CARs progressively (see Figure 2 that shows the diagram of the method steps). At each step, a set of potential adjacencies is first detected, then the method selects a subset of non-conflicting adjacencies that are added to the current CARs. The following three steps are used iteratively in order to collect the ancestral adjacencies: Step a) consists in detecting the conserved adjacencies and the homoplasy costs of these adjacencies are used to classify and select a subset of non-conflicting adjacencies to be added in current CARs; Step b) consists in resolving conflicts between adjacencies and selecting a subset of non-conflicting adjacencies to be added in current CARs; Step c) consists in detecting some adjacencies not conserved at the ancestral node, but supported by putative genome rearrangement events. In the next paragraphs, we briefly give an overview of each of these steps.

#### a) Adding non-conflicting conserved adjacencies

This step comes after the initialization phase, or after a step a), or b) or c) that ended up with a non-empty set of added adjacencies. The step begins with the detection of the conserved adjacencies between the current CARs at the ancestral node. Next, the non-conflicting fully-conserved adjacencies are selected in a first phase. Then, the non-conflicting partly-conserved adjacencies that are compatible with all fully-conserved adjacencies are added in a second phase. The set of all conserved adjacencies added in the CARs in this step is denoted by NC. It constitutes a non-conflicting set of adjacencies. The conserved adjacencies not added in this step are stored in a set C and tagged as conflicting adjacencies for a next step b).

#### b) Resolving conflicts between adjacencies

This step comes after a step a) that ended up with an empty set NC, and a non-empty set C. It considers the set C of adjacencies tagged as conflicting in this last step a). A cost, different from the homoplasy cost, is computed for each of these adjacencies, and a non-conflicting subset C*_m _*of C that has a maximum size and minimizes the sum of the adjacencies costs is computed. This subset of adjacencies is added in the CARs, and the remaining adjacencies of the set C−C*_m _*are discarded permanently.

#### c) Detecting DCJ-reliable adjacencies

This step comes after a step a) that ended up with an empty set NC, and an empty set C. It consists in finding new potential adjacencies that are not conserved at the ancestral node (*i.e*. neither partly-conserved nor fully-conserved). Each of these new potential adjacencies is supported by the presence of an adjacency in the current set of CARs, and two adjacencies in an extant genome *G*, such that those three adjacencies completed by the new potential adjacency induce a single genome rearrangement event, specifically Double-Cut-and-Join (DCJ) events, between the ancestral genome and the genome *G*. A maximum size non-conflicting subset of the new potential adjacencies is added in the CARs, and the remaining adjacencies are discarded permanently.

We now give the detailed descriptions of Step a), b) and c).

### Step a): Detection of non-conflicting conserved adjacencies

In this section, we first explain how the conserved adjacencies are defined. Next, we describe how a subset of non-conflicting adjacencies is selected by giving priority to the fully-conserved adjacencies.

#### Detection of the conserved adjacencies

We begin by stating the definition of a *CAR adjacency *in an extant genome at a leaf of the species tree, given the set of CARs in the current step of the method.

Let us recall that a *CAR *is an oriented sequence of signed blocks. We denote by |*x*| the block corresponding to a signed block *x *in a CAR. A *signed CAR *is a CAR possibly preceded by − indicating its reverse orientation. For example, if car_x = {*a −b c*}, then -car_x = {−*c b − a*}.

Let car_a and car_b be two signed CARs in the current set of CARs with car_a = {*a*_1 _*a*_2 _... *a_n_*} and car_b = {*b*_1 _*b*_2 _... *b_m_*}.

The ordered pair (car_a car_b) is a *CAR adjacency *in an extant genome *G *if there exists a pair of segments *S_a _*and *S_b _*consecutive in genome *G *such that the segment *S_a _*(resp. *S_b_*) contains only blocks from car_a (resp. car_b), and satisfies the following constraints:

1. i.) *S_a _*is either the segment {*a_n_*}, else ii) a segment of length *n_a _*> 1 ending with the block |*a_n_*|, else iii) a segment syntenic to a segment of car_a containing the block |*a_n_*|,

2. i) *S_b _*is either the segment {*b*_1_}, else ii) a segment of length *n_b _*> 1 starting with the block |*b*_1_|, else iii) a segment syntenic to a segment of car_b containing the block |*b*_1_|.

As for the blocks, the CAR adjacency (car_a car_b) is equivalent to (−car_b - car_a).

For example, consider the following three CARs composed of ten blocks:

car_1 = *• a b c • *;

car_2 = *• d e f g • *;

car_3 = *• h i j •*.

The genome *G *= *• b c −d f • *; *• e −g i j a −h • *has three CAR adjacencies: (car_1 car_2), (car_2 −car_3), and (car_3 car_1). The pair (car_1 car_2) is a CAR adjacency because of segment *S*_1 _= {*c*} and *S*_2 _= {*−d f *} that are consecutive in the genome *G*, and such that *S*_1 _satisfies the constraint 1.i) and *S*_2 _satisfies the constraint 2.ii). The CAR adjacency (car_2 - car_3) is supported by the segments *S*_2 _= {*e −g*} satisfying 1.ii) and *S*_3 _= {*i j*} = {*−j −i*} satisfying 2.iii). The CAR adjacency (car_3 car_1) is supported by the segments *S*_3 _= {*j*} satisfying 1.i) and *S*_1 _= {*a*} satisfying 2.i).

The *block adjacency corresponding to the CAR adjacency *(car_a car_b) with car_a = {*a*_1 _*a*_2 _... *a_n_*} and car_b = {*b*_1 _*b*_2 _... *b_m_*} is the adjacency (*a_n _b*_1_).

In the previous example, the block adjacencies corresponding to (car_1 car_2), (car_2 - car_3) and (car_3 car_1) are respectively (*c d*), (*g −j*), and (*j a*).

**Proposition 1 ***Let car_a *= {*a*_1 _*a*_2 _... *a_n_*} *be a signed CAR in the current set of CARs. An extant genome G has at most two CAR adjacencies of the form *(*car_a car_x*).

*Proof *Let us suppose that an extant genome *G *has more than two CAR adjacencies of the form (car_a car_x). Say (car_a car_x), (car_a car_y), and (car_a car_z) are three of them. These CAR adjacencies would be supported by 1) three pairs of consecutive segments on *G*, $\left(S_{a_{1}},S_{x}\right),\left(S_{a_{2}},S_{y}\right),\left(S_{a_{3}},S_{z}\right)$, such that 2) $S_{a_{1}},S_{a_{2}},S_{a_{3}}$ contain the block |*a_n_*|, and 3) *S_x_*, *S_y _*, *S_z _*are non-intersecting segments since they belong to three different CARs. It is impossible to find an ordering of the six segments on *G *such that the constraints 1), 2) and 3) are all satisfied simultaneously. Thus, the genome *G *contains at most two CAR adjacencies of the form (car_a car_x).   □

**Remark 1 ***The definitions of fully or partly conserved adjacencies are naturally extended to CAR adjacencies as follows: a *conserved CAR adjacency *at an ancestral node of the species tree is a CAR adjacency shared by at least two extant genomes that belong to at least two different sets of the species partition defined by the ancestral node. A *fully-conserved CAR adjacency *is a conserved CAR adjacency belonging to at least one genome of each of the three sets of the species partition defined by the ancestral node. A *partly-conserved CAR adjacency *is any other conserved CAR adjacency. The homoplasy cost associated to a CAR adjacency is a natural extension of the definition given for the block adjacencies*.

#### Classification and selection of the conserved adjacencies

The overall idea of this phase is to select conserved adjacencies while giving priority to the fully-conserved adjacencies, and to the adjacencies that have the less conflicts with other adjacencies.

**Figure 3:**
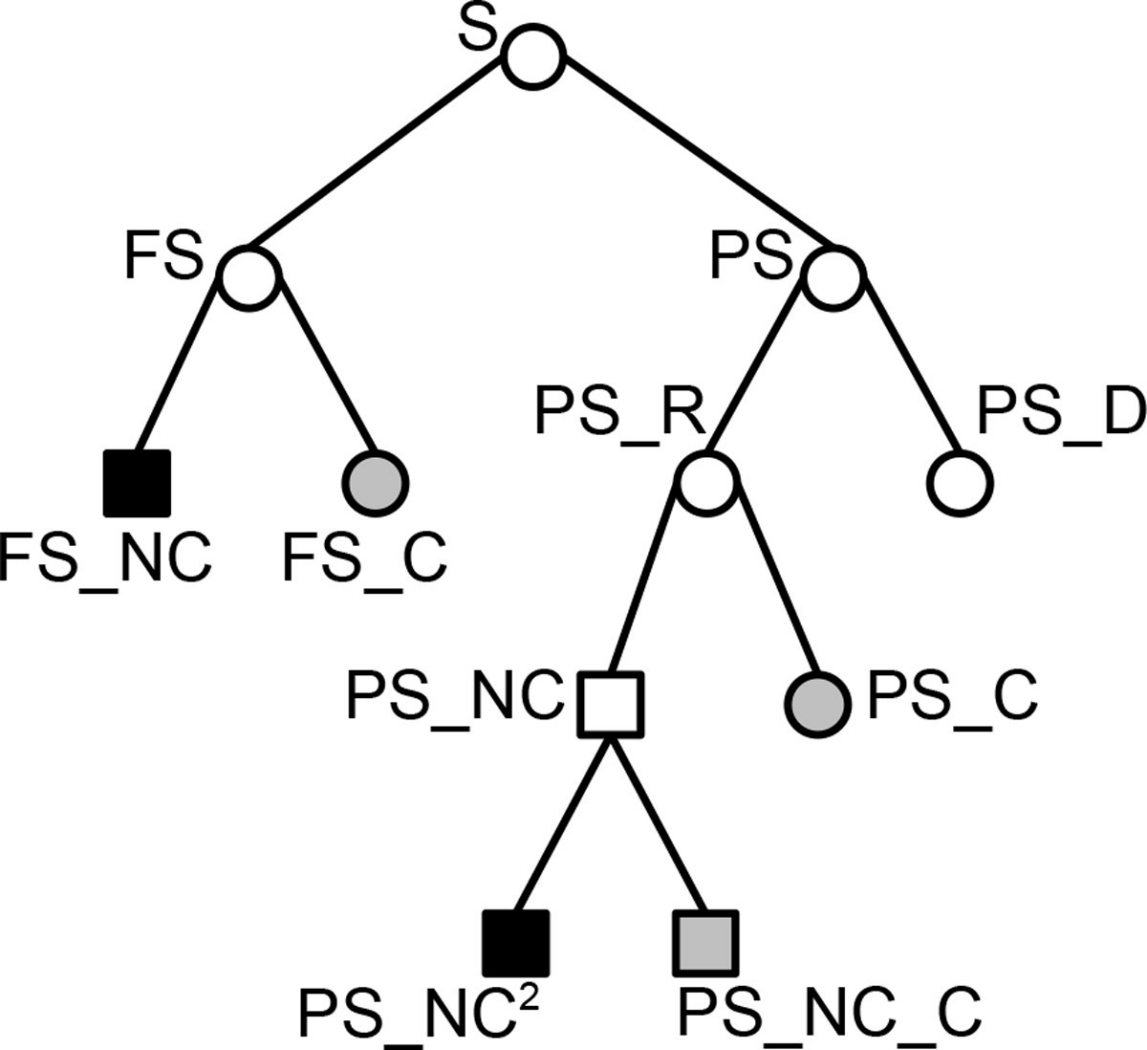
**Organization of the sets of adjacencies considered in Step a)**. A tree whose nodes represent sets of conserved adjacencies found at the current step of the method, and edges represent the inclusion relations between the sets: the root of the tree is the set S of all conserved adjacencies. Abbreviations: FS (Fully-conserved adjacencies), PS (Partly-conserved adjacencies), NC (Non-Conflicting), C (Conflicting), R (Retained), and D (Discarded). Non-conflicting sets are represented with square nodes. The sets of non-conflicting adjacencies added at the current step are represented with black-colored nodes. The sets of conflicting adjacencies saved for the next step b) are represented with gray-colored nodes.

Let S be the set of block adjacencies corresponding to the conserved CAR adjacencies at the ancestral node. In the sequel, the abbreviations FS, PS, NC, C stand for *Fully*, *Partly*, *Non-Conflicting*, and *Conflicting *conserved adjacencies respectively. Figure 3 shows the organization of the sets of adjacencies that are considered in this phase.

Let FS and PS be the subsets of S that contain respectively the fully-conserved adjacencies and the partly-conserved adjacencies. Thus, *S *= FS ∪ PS and FS *∩ *PS = ∅.

First, we consider the fully-conserved adjacencies. Let FS_NC be the subset of FS that contains the adjacencies that are compatible with all other adjacencies in FS. The corresponding set of conflicting adjacencies is FS_C = FS - FS_NC. The fully-conserved non-conflicting adjacencies contained in the set FS_NC are automatically retained to be added in the CARs. Thus, (*) in the following, any adjacency that is in conflict with some adjacencies of FS_NC will be discarded permanently.

Next, we consider the partly-conserved adjacencies. Let PS_D be the subset of PS containing adjacencies that are in conflict with some adjacencies of FS_NC, and PS_R be the set of the remaining adjacencies in PS. Thus, PS_R = PS - PS_D. The adjacencies of PS_D are discarded permanently, as explained previously in (*).

Let PS_NC be the subset of PS_R that contains the adjacencies that are compatible with all other adjacencies in PS_R. The corresponding set of conflicting adjacencies is PS_C = PS_R - PS_NC.

Finally, since the priority is given to fully-conserved adjacencies, we want to retain only the adjacencies of PS_NC that are not in conflict with the adjacencies of the set FS. Let PS_NC^2 ^be the subset of PS_NC that contains the adjacencies that are compatible with all the adjacencies in FS. The partly-conserved non-conflicting adjacencies contained in the set PS_NC^2 ^are also retained automatically to be added in the CARs.

It follows that the set of retained adjacencies NC = FS_NC ∪ PS_NC^2 ^is a set of non-conflicting adjacencies.

This step a) of the method adds the set of adjacencies NC to the current CARs of the ancestral genome, and updates the current set of conflicting adjacencies to the set C = S - PS_D − NC. By construction, each adjacency contained in the set C is in conflict with at least one other adjacency of C, and compatible with all the adjacencies contained in the set NC.

The step a) can be recalled several times consecutively as far as the set NC of added adjacencies is not empty. We now state a proposition ensuring that the current set of conflicting adjacencies *C *misses no previously found conflicting adjacency (*a b*) such that the signed block *a *is the end of a signed CAR, and the signed block *b *is the start of a signed CAR in the current set of CARs.

**Proposition 2 ***Let *(*a b*) *be an adjacency corresponding to a conserved CAR adjacency found in a previous step a) of the method. The adjacency *(*a b*) *is either present in the current set of CARs, or is in conflict with an adjacency present in the current set of CARs, or is also found in the current step a) i.e *(*a b*) ∈ *S*.

*Proof *Say that, in a previous step a), the adjacency (*a b*) was supported by the detection of a conserved CAR adjacency (car_a_1 _car_b_1_) present in a subset *G *of the extant genomes.

1) If there exist in the current set of CARs, a signed CAR car_a_2 _ending with the signed block *a*, and a signed CAR car_b_2 _starting with the signed block *b*, then the CAR adjacency (car_a_2 _car_b_2_) is also found in the same set  G of extant genomes. Thus, the adjacency (*a b*) is also found in the current step.

2) Otherwise, either there exists an adjacency of the form (*a c*) or (*c b*) in the current set of CARs in conflict with the adjacency (*a b*), or the adjacency (*a b*) is present in the current set of CARs.   □

### Step b): Resolution of conflicts between adjacencies

This step considers a conflicting set C of adjacencies obtained at the end of a previous step a), and computes a non-conflicting subset of the set C to be added in the current set of CARs.

#### Definition of the cost of adjacencies

We begin by stating the definition of the cost of an adjacency in this step. The *mutation cost *of a labeling of the nodes of a species tree on a given alphabet is the number of edges in the tree having two different labels at their extremities [13,14]. Here, the *cost of an adjacency *(*a b*) ∈ C is the minimum mutation cost of a labeling of the nodes of the species tree on a binary alphabet {0, 1} such that (i) the ancestral node is labeled with 1, (ii) the extant species nodes, where (*a b*) corresponds to a CAR adjacency, are labeled with 1, and (iii) the other extant species nodes are labeled with 0.

In other terms, an adjacency has two possible states in a genome: present (1) or absent (0). The cost of an adjacency (*a b*) is the minimum number of changes of state necessary to explain the evolutionary history of the adjacency along the species tree, with the adjacency being present at the ancestral node.

**Figure 4:**
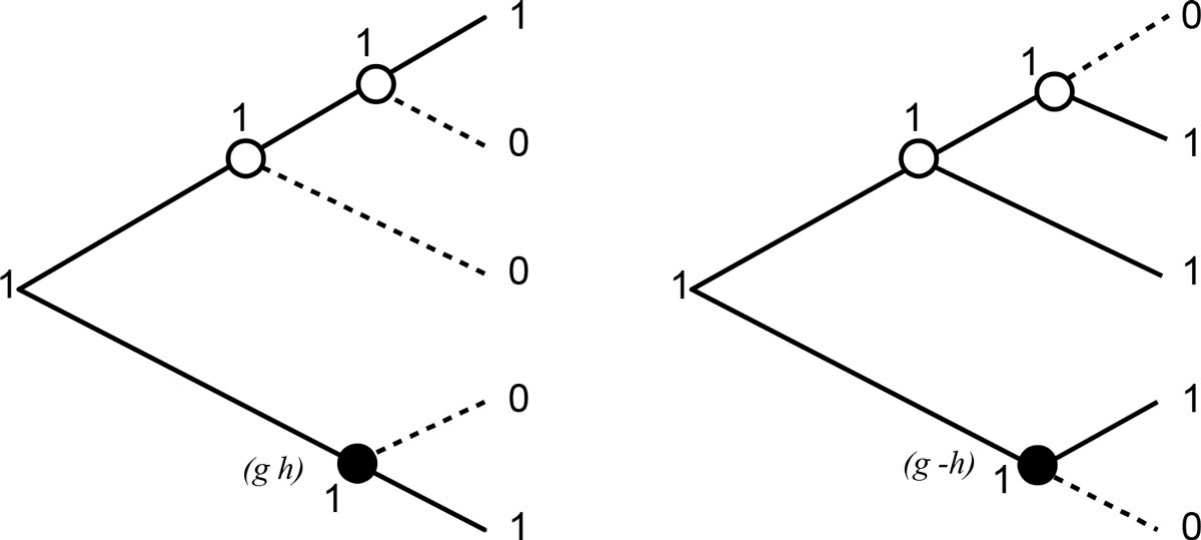
**Examples of minimum mutation cost labelings of the nodes of a species tree**. Right and left trees show two minimum mutation cost labelings of the nodes of the species tree for the adjacencies (*g h*) (left labeling) and (*g −h*) (right labeling) conserved at the ancestral node in the species tree depicted in Figure 1. For each labeling, the edges of tree with a change of state are in dashed line. The cost of the left labeling is 3, and the cost of the right labeling is 2.

For example, the costs of the two conflicting conserved adjacencies (*g h*) and (*g −h*) shown in Figure 1 are 3 and 2 respectively. Figure 4 shows two minimum mutation cost labelings of the nodes of the species tree corresponding to both adjacencies.

#### Computation of the non-conflicting subset of adjacencies

The *cost of a set of adjacencies *is the sum of the costs of the adjacencies composing this set.

Let *m *be the maximum size of a non-conflicting subset of the conflicting set C of adjacencies. This step b) finds a non-conflicting subset C*_m _*of C of size *m *and minimum cost. The set of adjacencies C*_m _*is added to the current CARs of the ancestral genome, and the remaining adjacencies in the set C − C*_m _*are discarded permanently.

**Remark 2 ***Note that the adjacencies of the set C − C_m _discarded in this step will never be detected again, since these adjacencies are in conflict with the adjacencies of the set C_m _added in the current step*.

### Step c): Detection of DCJ-reliable adjacencies

This step considers the current set of CARs, and computes new potential adjacencies not conserved, but supported by putative ancestral rearrangement events.

A *Double-Cut-and-Join (DCJ) rearrangement event *on a genome consists in the cut of two adjacencies of the genome in order to glue the four exposed extremities in a different way. For example, a DCJ event on the genome *A *= (*• a b c d •*) that cuts the adjacencies (*a b*) and (*c d*) to obtain the adjacencies (*a −c*) and (*−b d*) produces the genome *B *= (*• a −c −b d •*).

We now give the definition of potential ancestral adjacencies that can be inferred from putative genome rearrangements inspired from the definitions of reliable adjacencies in [15,16].

Here, we add the constraint that the signal of the reliable adjacency must be conserved on a path of the species tree that goes through the ancestor.

Let car_a and car_b be two signed CARs in the current set of CARs with car_a = {*a*_1 _*a*_2 _... *a_n_*} and car_b = {*b*_1 _*b*_2 _... *b_m_*}. The adjacency (*a_n _b*_1_) is a *DCJ-reliable adjacency *of the ancestral node if there exists an adjacency (*x y*) in the current set of CARs such that the adjacencies (*x b*_1_) and (*a_n _y*) are present in an extant genome *G*_1_, and (car_a car_b) is a CAR adjacency in an extant genome *G*_2 _such that the genomes *G*_1 _and *G*_2 _belong to two different sets of the species partition defined by the ancestral node.

The potential presence of the adjacency (*a_n _b*_1_) in the ancestral genome induces a DCJ event that has cut the adjacencies (*a_n _b*_1_) and (*x y*) in the ancestral genome to produce the adjacencies (*x b*_1_) and (*a_n _y*) in the extant genome *G*_1_.

An example is given in Section Results and discussion.

In this step of the method, a maximum size non-conflicting subset of the DCJ-reliable adjacencies is added in the CARs, and the remaining DCJ-reliable adjacencies are discarded permanently.

**Remark 3 ***Note that the homoplasy cost of a DCJ reliable adjacency is always *2.

## Results and discussion

We used ProCARs to compute a set of CARs for the boreoeutherian ancestral genome using the block orders of twelve amniote genomes, and we compared the result with the ancestors reconstructed by three other homology-based methods: AnGeS [12], InferCARs [5] and GapAdj [11].

### Orthology blocks and phylogeny

We chose twelve genomes completely assembled and present in a Pecan [17] multiple alignment of 20 amniote genomes available in the release 73 of the Ensembl Compara database [18]. The phylogenetic tree was directly infered from the classifications of the species obtained from the National Center for Biotechnology Information Taxonomy database [19] (see Additional File 1). We constructed a set of synteny blocks using the multiple alignments as seeds. We used the block construction method described in [20], keeping only the seeds that had an occurrence in each of the twelve genomes, removing the seeds that spanned less than 100Kb, and joining seeds collinear in all genomes. This resulted in a set of 12 genomes composed of 689 blocks for species *Homo sapiens *(GRCh37), *Pan troglodytes *(CHIMP2.1.4), *Pongo abelii *(PPYG2), *Macaca mulatta *(MMUL 1), *Mus musculus *(GRCm38), *Rattus norvegicus *(Rnor 5.0), *Equus caballus *(EquCab2), *Canis familiaris *(CanFam3.1), *Bos taurus *(UMD3.1), *Monodelphis domestica *(BROADO5), *Gallus gallus *(Galgal4) and *Taeniopygia guttata *(taeGut3.2.4).

### Reconstruction of the boreoeutherian ancestor

ProCARs ran in 5 steps and finally returned 25 CARs with a number of blocks per CAR ranging from 2 to 68 (Table 1). The total number of adjacencies computed for the boreoeutherian ancestor is 664 compared to the 666, 669, 659 adjacencies present in respectively *Homo sapiens*, *Mus musculus *and *Bos taurus*.

**Table 1:** Steps of ProCARs.

Step	0: init	1: step a)	2: step a)	3: step b)	4: step c)	5 step a)
#CARs	689	45	32	30	27	25

size	1	1 - 67	1 - 68	1 - 68	2 - 68	2 - 68

#adjacencies	0	647	9	3	3	2

Number of CARs, number of blocks per CAR, and number of new adjacencies returned at each iteration of ProCARs method.

The numbers of blocks per CAR are detailed in Table 2. The human chromosomal syntenies are 1-5, 3-21, 4-8, 8-19, 12-22, 14-15 and 16-19. In [21], the boreoeuthe-rian ancestor has two more human chromosomal syntenies 7-16 and 10-12-22, and all other syntenies were also found by ProCARs.

**Table 2:** CARs of ProCARs.

CAR	1	2	3	4	5	6	7	8	9	10	11	12	
size	57	46	9	27	36	3	17	15	53	12	18	32	

hcs	1	**1-5**	10	10	11	12	**12-22**	13	**14-15**	16	**16-19**	17	

CAR	13	14	15	16	17	18	19	20	21	22	23	24	25

size	20	15	28	30	28	68	50	43	7	20	2	47	6

hcs	18	**8-19**	2	2	20	**3-21**	**4-8**	6	7	7	8	9	X

Number of blocks and human chromosomal syntenies (hcs) for each CAR computed by ProCARs. Human chromosomal syntenies involving two human chromosomes are in bold.

### Comparison with other methods

All the methods (ProCARs, AnGeS [12], InferCARs [5] and GapAdj [11]) take as input a phylogeny with a tagged ancestral node in this phylogeny, and a set of blocks with the arrangement of the blocks in each extant genome of the phylogeny. AnGeS [12] first builds a set of potential ancestral features (adjacencies, and sets of contiguous blocks) by comparing pairs of species whose path goes through the tagged ancestral node. Then, an arrangement of the blocks that corresponds to a subset of these adjacencies is built in order to satisfy the consecutive ones property. This assembling phase requires the length of the branches of the phylogenetic tree. InferCARs [5] is based on an adaptation of the Fitch parsimony method for adjacencies. Potential neighbors of blocks are modeled through graphs at each node of the phylogenetic tree. Conflicts between potential neighboring relations are resolved using a weight function which requires the length of the branches of the phylogenetic tree. GapAdj [11] works iteratively, detecting new adjacencies at each step by allowing more and more gaps within adjacencies until the maximum number of gaps MAX*_α _*is reached. At each step, the assembling of the extended CARs is done using a TSP algorithm, and a threshold *τ *is required to discard the less reliable adjacencies.

As GapAdj is the only method with parameters (MAX*_α _*and *τ *), we ran GapAdj on 500 sets of parameters for MAX*_α _*ranging from 1 to 10, and *τ *ranging from 0.50 to 0.99. We then selected the reconstruction that had the minimal breakpoint distance to the ancestor reconstructed by ProCARs. The breakpoint distance between two genomes is the number of blocks extremities whose neighbors are not conserved in both genomes. Among the 500 sets of parameters tested, the closest result is obtained when *τ *equals 0.79 and MAX*_α _*equals 3, giving a breakpoint distance of 32.5 between this reconstruction and the ancestor reconstructed by ProCARs. That corresponds to 4.7% of the block extremities having different neighbors in both reconstructions. Note that the reconstruction selected for GapAdj is also the closest to the ancestors reconstructed by InferCARs and AnGeS.

**Figure 5:**
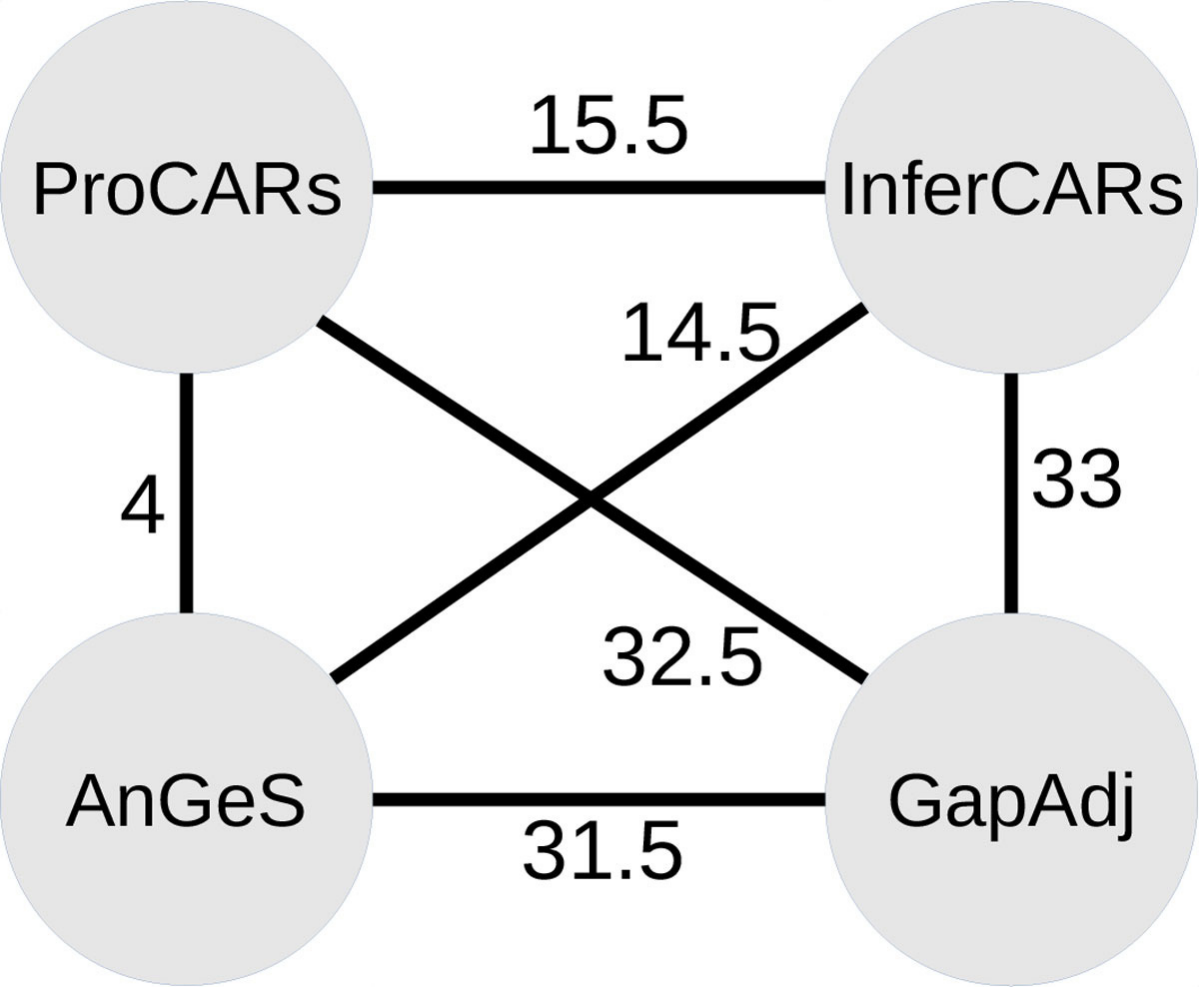
**Breakpoint distances between the sets of CARs**. The label on each edge gives the breakpoint distance between the two methods in the nodes.

**Figure 6:**
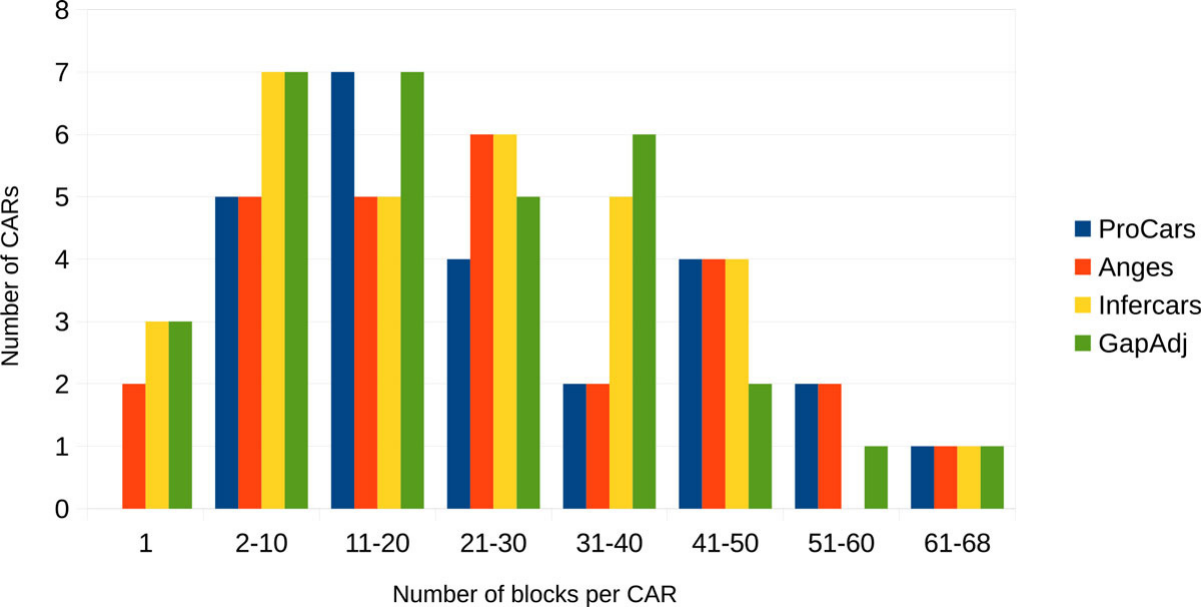
**Distribution of the number of blocks involved in each CAR**. For each of the four methods, the number of CARs for which the number of blocks is in a given range is plotted.

Figure 5 gives the breakpoint distances between all pairwise comparisons. This shows that GapAdj is the method which gives the most different result, while AnGeS is the method which finds the closest result to ProCARs. The distribution of the number of blocks involved in each CAR is roughly the same (Figure 6).

**Figure 7:**
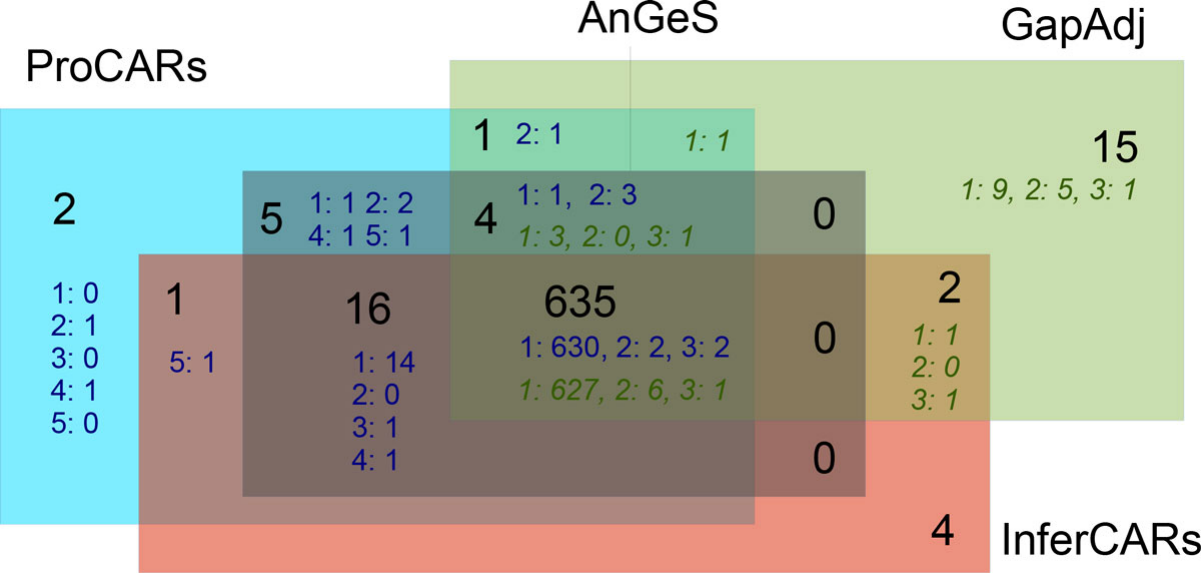
**Number of adjacencies shared or exclusive for each of the four methods compared**. AnGeS contains no specific adjacency. For example, 635 adjacencies are shared by all the methods, and 16 are shared between AnGeS, InferCARs and ProCARs. For ProCARs and GapAdj (in italic), we also give the number of adjacencies and the step in which they have been added. For example, there are 15 adjacencies exclusive to GapAdj, of which 9 have been added at step 1, 5 at step 2 and 1 at step 3.

We then computed the list of adjacencies shared by all the methods and the adjacencies that are specific to a subset of the methods, as shown in Figure 7. The number of adjacencies shared by all the methods is 635. The method which infers the highest number of specific adjacencies is GapAdj (15 adjacencies), as suggested by the breakpoint distances shown in Figure 5. All adjacencies found by AnGeS are also found by ProCARs, as confirmed by the small breakpoint distance between the two methods. Finally, it is noteworthy that there is no adjacency shared by the three methods AnGeS, InferCARs and GapAdj that is not found by ProCARs.

#### Justification of ProCARs specific adjacencies

Adjacency (50 − 545) is found at iteration 4 of ProCARs. In the other methods, the block 50 is alone in a single-block CAR. This adjacency was detected thanks to a step c) of ProCARs method. It is then a DCJ-reliable adjacency detected as follows: at iteration 3, adjacency (535 536) was found, block 50 is at the end of CAR 2, and block 545 is at the end of CAR 20. In *Bos taurus*, the CAR adjacency (2 − 20) is conserved. In *Mus musculus*, block adcencies (545 536) and (50 − 535) are present. Hence, as the path from *Bos taurus *to *Mus musculus *goes through the ancestor, a potential DCJ rearrangement on adjacencies (50 − 545) and (535 536) in the ancestor could explain the adjacencies found in *Mus musculus*: (545 536) and (50 − 535). Moreover, this adjacency (50 − 545) found by ProCARs is the one supporting the human chromosomal synteny 1-5 that was also reported in [21] using a cytogenetic method, but not found by any of the other methods (see Table 3).

**Table 3:** Comparison of human chromosomal syntenies.

Human chromosomal syntenies	1-5	3-21	4-8	7-16	8-19	12-22	14-15	16-19	7-9	5-6-18	10-11
In [21]	*•*	*•*	*•*	*•*	*•*	+10	*•*	*•*	--	--	--

ProCARs	*•*	*•*	*•*	--	*•*	*•*	*•*	*•*	--	--	--

AnGeS	--	*•*	*•*	--	*•*	*•*	*•*	*•*	--	--	--

InferCARs	--	*•*	*•*	--	--	*•*	--	--	--	--	--

GapAdj	--	*•*	+2	--	*•*	*•*	*•*	--	*•*	*•*	*•*

For each method, we give which human chromosomal synteny is found. A number in a cell indicates that the synteny is found but with additionnal part of another chromosome.

Adjacency (616 618) is found at iteration 2. It is then a conserved adjacency detected as follows: at iteration 1, block 616 is alone in a CAR, and block −618 is at the end of a CAR. This adjacency (616 618) is present, on the one hand, in *Mus musculus *and *Rattus norvegicus *(ingroup *I*_2_) and on the other hand in *Equus caballus *and *Bos taurus *(ingroup *I*_1_). Hence, it is a partly-conserved adjacency and, as it is not in conflict with any other conserved adjacency, ProCARs joined 616 and 618 at iteration 2. In InferCARs and AnGeS, 616 is alone in a CAR while 618 is also at the begining of a CAR. Therefore, CARs found by InferCARs and AnGeS are not in conflict with the adjacency (616 618) that ProCARs added, but no signal was found by those methods to infer this adjacency. In GapAdj, 618 is alone in a CAR while 616 is in a CAR containing (−299 616 617). However, (616 617) is only present in species in the ingroup *I*_2 _(*Homo sapiens*, *Pan troglodytes*, *Pongo abelii *and *Macaca mulatta*). Therefore it is not a conserved adjacency, and that is why ProCARs preferred the partly-conserved adjacency (616 618).

#### Justification of adjacencies not found by ProCARs

AnGeS contains no specific adjacency and thus ProCARs found all adjacencies detected by AnGeS. There are 2 adjacencies found by both GapAdj and InferCARs but not by ProCARs.

For adjacency (67 68), InferCARs inferred a unique CAR which is the concatenation of CARs 3 and 4 of ProCARs involving respectively blocks 67 and 68. GapAdj also inferred this concatenation of the two ProCARs CARs, except a segment of the CAR involving block 68 in ProCARs which is in a separated CAR. The (67 68) adjacency is only present in *Homo sapiens*, *Pan troglodytes*, *Pongo abelii *and *Macaca mulatta *(ingroup *I*_2_) and hence cannot be a partly-conserved adjacency. It is not a DCJ-reliable adjacency either.

Concerning the adjacency (−657 658), ProCARs has adjacencies (−657 − 659 − 658) in CAR 24 while InferCARs (resp. GapAdj) created adjacencies (−657 658 659) in CAR 28 (resp. 27). The adjacency (−657 658) is present only in *Mus musculus *and *Rattus norvegicus *(ingroup *I*_2_) and is hence not a conserved adjacency. It is not a DCJ-reliable adjacency either, otherwise this adjacency would have been detected during iteration 4.

#### Human chromosomal syntenies

Human syntenies found by other methods are: for AnGeS: 3-21, 4-8, 8-19, 12-22, 14-15, 16-19; for InferCARs: 3-21, 4-8 and 12-22; for GapAdj: 2-4-8, 3-21, 7-9, 5-6-18, 8-19, 10-11, 12-22 and 16-19. A comparison between the four methods is given in Table 3 and a karyotype of the ancestral genomes in Additional File 2. We can notice that ProCARs returns the closest result to the ancestor reconstructed in [21] using a cytogenetic method.

## Conclusions

InferCARs is the first method using an adaptation of the Fitch algorithm to infer ancestral gene orders based on homology instead of rearrangements. AnGeS makes use of common intervals to be able to account for micro-rearrangements. GapAdj brings the iterative approach allowing to build CARs step by step. With ProCARs, we propose a new methodology which combines the different approaches found in other methods, using a model based on adjacencies only.

ProCARs has the advantage to be a parameter-free method, without the requirement of branch lengths for the phylogenetic tree. ProCARs is based on a single definition of contiguity, the CAR adjacency, that allows some micro-rearrangements under a very simple model. However, since ProCARs considers only genomes containing the same set of non-duplicated blocks, it does not allow to reconstruct ancestors in the context of duplication or loss events.

In order to select the adjacencies at each step of ProCARs, the adjacencies are classified according to an homoplasy cost instead of using a heuristic assembly algorithm. ProCARs gives priority to discarding conflicting adjacencies rather than necessarily adding new adjacencies at each step.

The final result of ProCARs is a set of completely resolved CARs, for which the arrangements of all the blocks are given.

As for other homology-based methods, ProCARs is not suitable in the case of convergent evolution. ProCARs is also a greedy algorithm which could be seen as a disadvantage because adjacencies are added permanently at each step. However, this greediness is balanced by the fact that ProCARs works iteratively and adds only reliable non-conflicting adjacencies at each step.

## Availability of supporting data

ProCARs is written in Python and is available at http://bioinfo.lifl.fr/procars. The dataset used in section

Results and discussion is also available from this web page.

## List of abbreviations

TSP: Traveling Salesman Problem; CAR: Contiguous Ancestral Region; NC: Non-Conflicting adjacencies; C: Conflicting adjacencies; FS: Fully-conserved adjacencies; PS: Partly-conserved adjacencies; R: Retained adjacencies; D: Discarded adjacencies.

## Competing interests

The authors declare that they have no competing interests.

## Authors' contributions

AP wrote the program and its documentation, ran the experiments, and presented the results at the conference ISCB-LA'14. AP, JSV, SB and AO analyzed and interpreted the data. JSV wrote the results section. AO conceived the study and its design, wrote a prototype of the program, and drafted the manuscript. AP, JSV and SB critically revised the manuscript. All authors read and approved the final manuscript.

## Supplementary Material

Additional file 1 **Phylogeny of the 12 species used in the application**. A figure at the PDF format depicting the phylogeny of the 12 species used in the application. The black node in the phylogeny corresponds to the boreoeutherian ancestor. Click here for file

Additional file 2 **Chromosomal syntenies with the human genome**. A figure at the PDF format depicting the Human chromosomal syntenies between the boreoeutherian ancestor found by the four methods ProCARs, InferCARs, GapAdj and AnGeS. Click here for file
