# Supplementary figures and images for: ProCARs: Progressive Reconstruction of Ancestral Gene Orders
Source: BMC Genomics. 2015 May 26;16(Suppl 5):S6. doi: 10.1186/1471-2164-16-S5-S6 (PMC4460626; doi:10.1186/1471-2164-16-S5-S6)

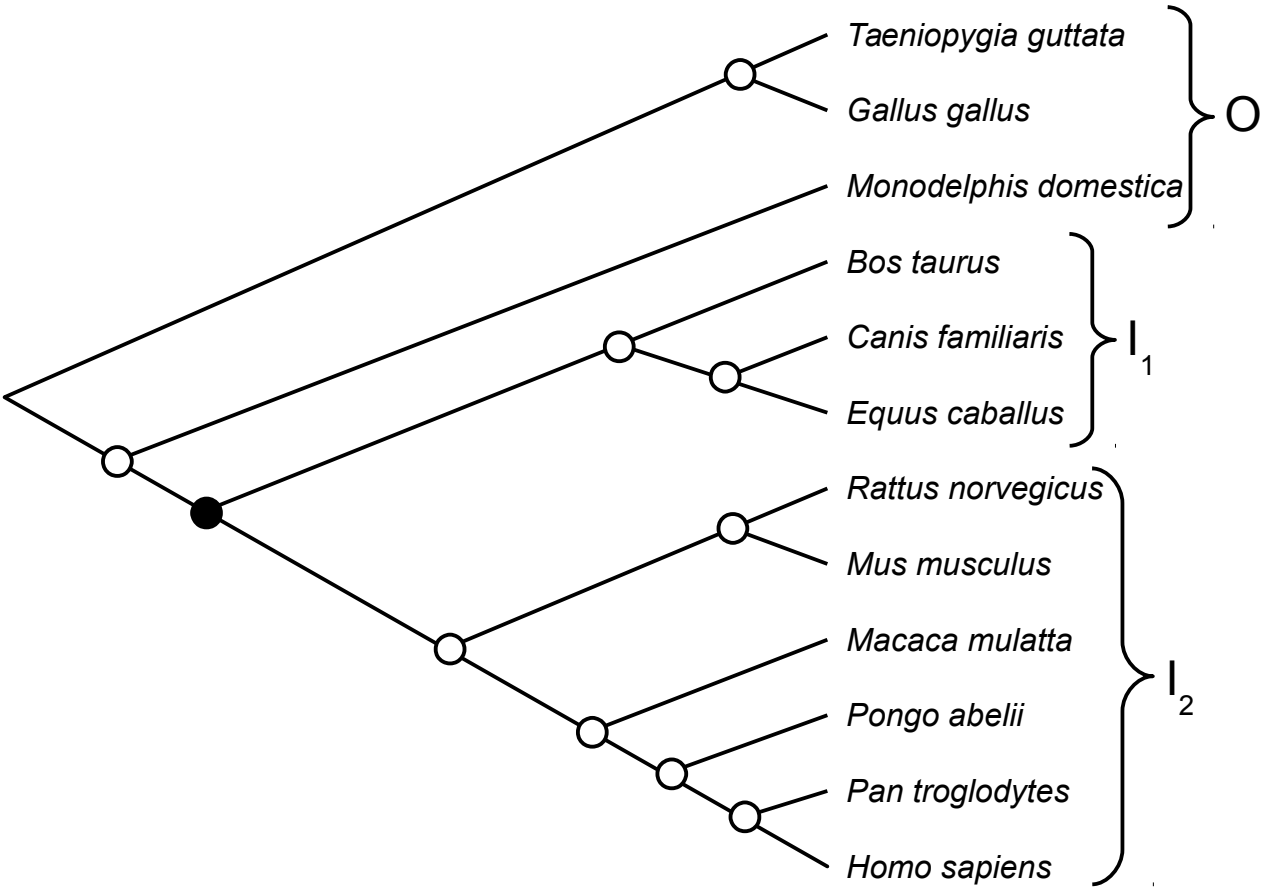

Supplement: Additional file 1 — Phylogeny of the 12 species used in the application. A figure at the PDF format depicting the phylogeny of the 12 species used in the application. The black node in the phylogeny corresponds to the boreoeutherian ancestor. [file 1471-2164-16-S5-S6-S1.pdf]

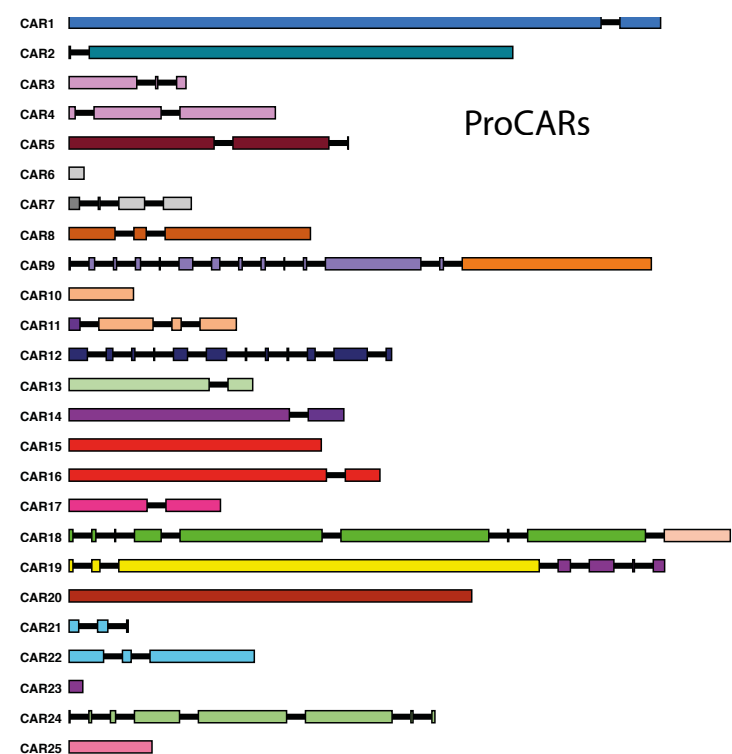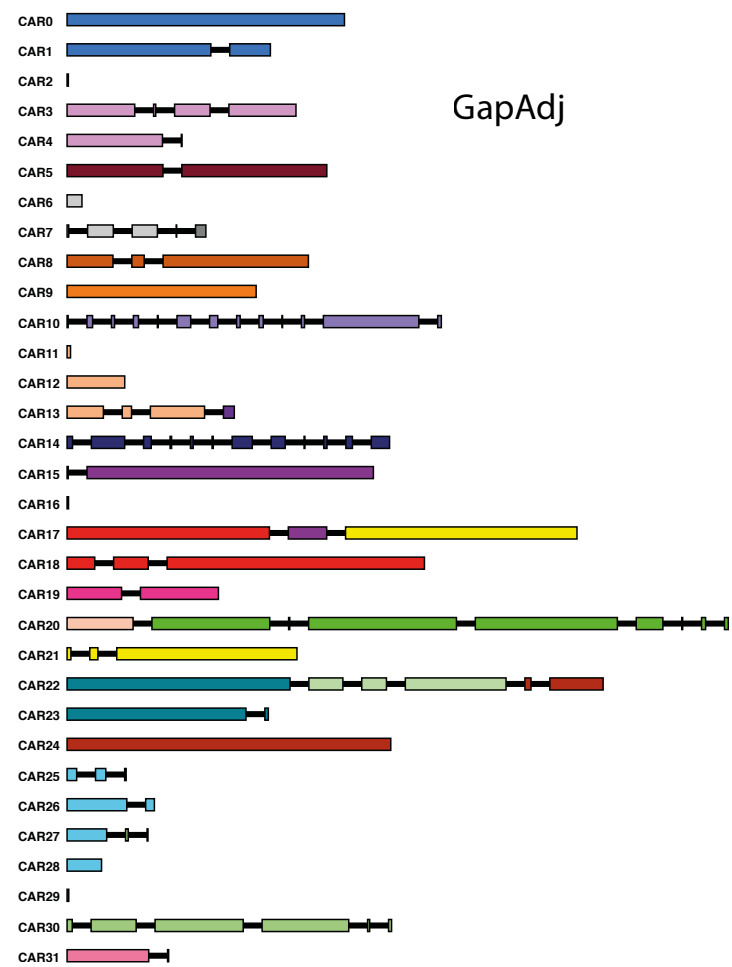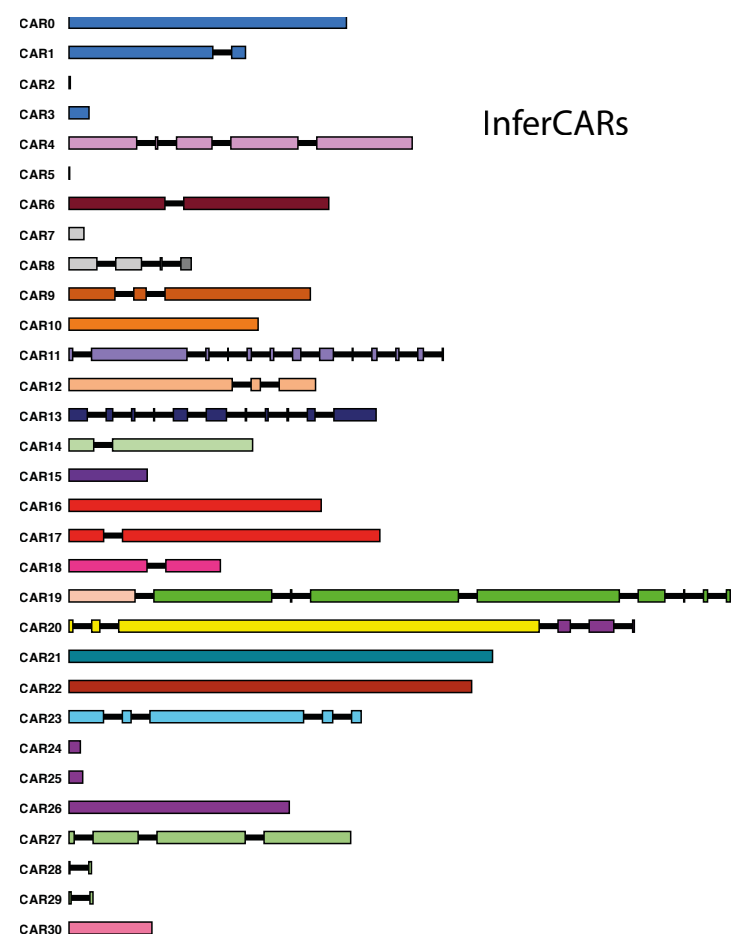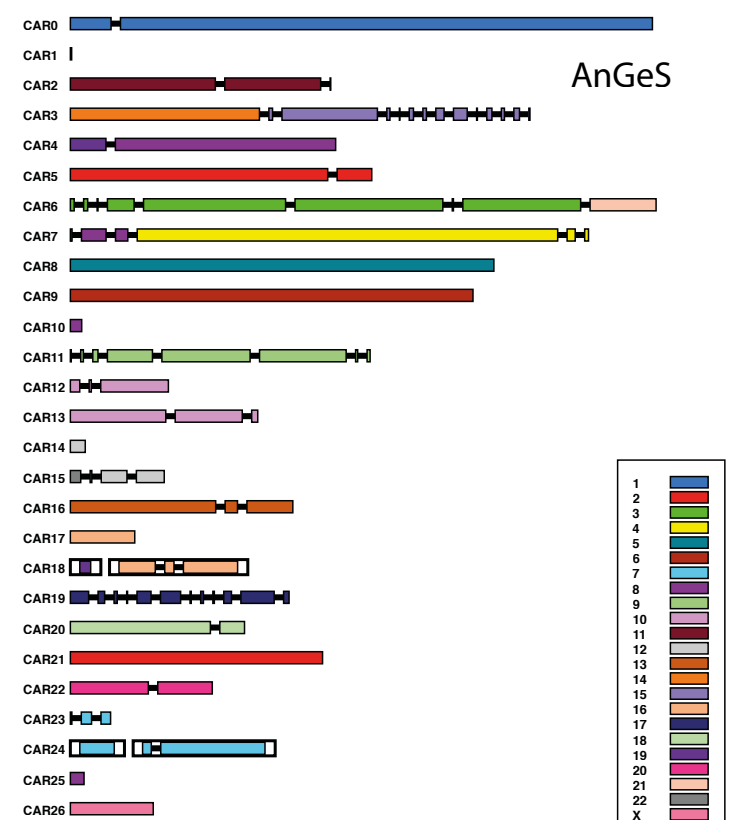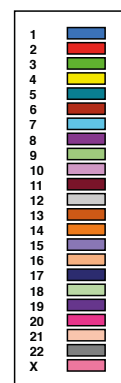

Supplement: Additional file 2 — Chromosomal syntenies with the human genome. A figure at the PDF format depicting the Human chromosomal syntenies between the boreoeutherian ancestor found by the four methods ProCARs, InferCARs, GapAdj and AnGeS. [file 1471-2164-16-S5-S6-S2.pdf]
